# Door-to-Balloon Time Delay in Complex Primary Angioplasty: A Case of Anomalous Origin of the Right Coronary Artery From the Pulmonary Artery (ARCAPA)

**DOI:** 10.1155/cric/6420460

**Published:** 2025-10-23

**Authors:** Giulio Montefusco, Luca Arcari, Salvatore Donato Musarò, Giovanni Camastra, Francesco Marino, Bruno Pironi, Francesca Fanisio, Massimiliano Danti, Stefano Sbarbati, Luca Cacciotti

**Affiliations:** ^1^Department of Emergency Medicine, Cardiology Unit, Madre Giuseppina Vannini Hospital, Rome, Italy; ^2^Department of Clinical, Internal, Anesthesiology and Cardiovascular Sciences, La Sapienza University of Rome, Rome, Italy; ^3^Department of Medicine, Cardiology Unit, Policlinico Casilino Hospital, Rome, Italy; ^4^Department of Emergency Medicine, Radiology Unit, Madre Giuseppina Vannini Hospital, Rome, Italy

**Keywords:** acute coronary syndrome, ARCAPA, congenital coronary anomalies, door-to-balloon time, multimodality imaging

## Abstract

Anomalous origin of the right coronary artery from the pulmonary artery (ARCAPA) is a rare condition, with clinical presentations ranging from sudden cardiac death to heart failure, although most cases remain asymptomatic until adulthood. We report the case of a 58-year-old man who presented to the emergency department with chest pain and persistent ST-segment elevation in the lateral leads (DI and aVL) on electrocardiogram (EKG). Coronary angiography revealed a subocclusive stenosis of the ramus intermedius (RIA) of the left coronary artery. Failure to selectively engage the RCA during the procedure, together with visualization of collateral circulation on aortography, raised suspicion of ARCAPA. Primary angioplasty was performed on the RIA with the implantation of a biodegradable polymer biolimus-eluting stent. The presence of ARCAPA resulted in approximately a 20% increase in door-to-balloon time compared with the institutional median of 65 min. The anomaly was subsequently confirmed by coronary computed tomography angiography (CCTA). This case highlights that coronary anomalies can increase the complexity of percutaneous coronary intervention (PCI) for ST-elevation myocardial infarction, prolong door-to-balloon times, and require interventional cardiologists to maintain a high level of awareness, understanding, and preparedness for such conditions.

**Summary**

• To emphasize the comprehension of various coronary artery anomalies and the effective treatment strategies for their management to avoid door-to-balloon time delays in primary angioplasty.
• To underline the pathophysiology of myocardial ischemia mechanisms resulting from congenital coronary artery anomalies.

## 1. Introduction

The majority of coronary anomalies manifest during infancy and represent a significant cause of myocardial ischemia and heart failure in the pediatric population. Anomalous origin of the right coronary artery from the pulmonary artery (ARCAPA) is a rare coronary anomaly that affects approximately 0.002% or 0.12% of all congenital heart disease (CHD) cases [1]. The clinical manifestations and timing of symptom onset depend on the development of collateral circulation following the decline in pulmonary artery pressure (PAP) and pulmonary vascular resistance (PVR) during the first weeks of life. Here, we report the case of a 58-year-old man with lateral persistent ST-elevation myocardial infarction (STEMI) presentation in which unknown ARCAPA led to a challenging catheterization during primary coronary angioplasty thus increasing the door-to-balloon time.

## 2. Case Report

**Figure 1:**
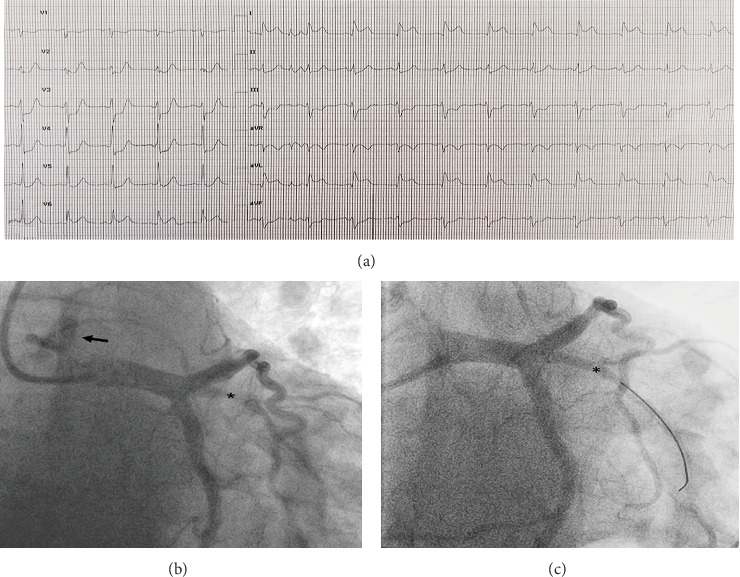
(a) A 12-lead electrocardiogram on admission shows ST-segment elevation in lateral leads (DI and aVL). (b, c) Coronary angiography, RAO caudal view: before (b) and after (c) PCI of the subocclusive stenosis (marked with an asterisk) of the proximal portion of the ramus intermedius artery (RIA). Retrograde filling of the right hypoplastic coronary artery toward the pulmonary artery (arrow in b).

A 58-year-old man presented to the emergency department with sweating and chest pain that had started 90 min earlier. He did not have a past medical history. Physical examination showed chest pain and no clinical signs of heart failure (Killip Class I). A 12-lead electrocardiogram (EKG) at admission revealed sinus rhythm with persistent ST elevation in lateral leads (DI and aVL) (Figure 1a). A transthoracic echocardiogram (TTE) showed preserved left ventricular ejection fraction (LVEF 50%) with lateral and inferolateral left ventricular wall hypokinesia. No significant valvular dysfunction and no pericardial effusion were found (Supporting Information 1: Video S1).

**Figure 2:**
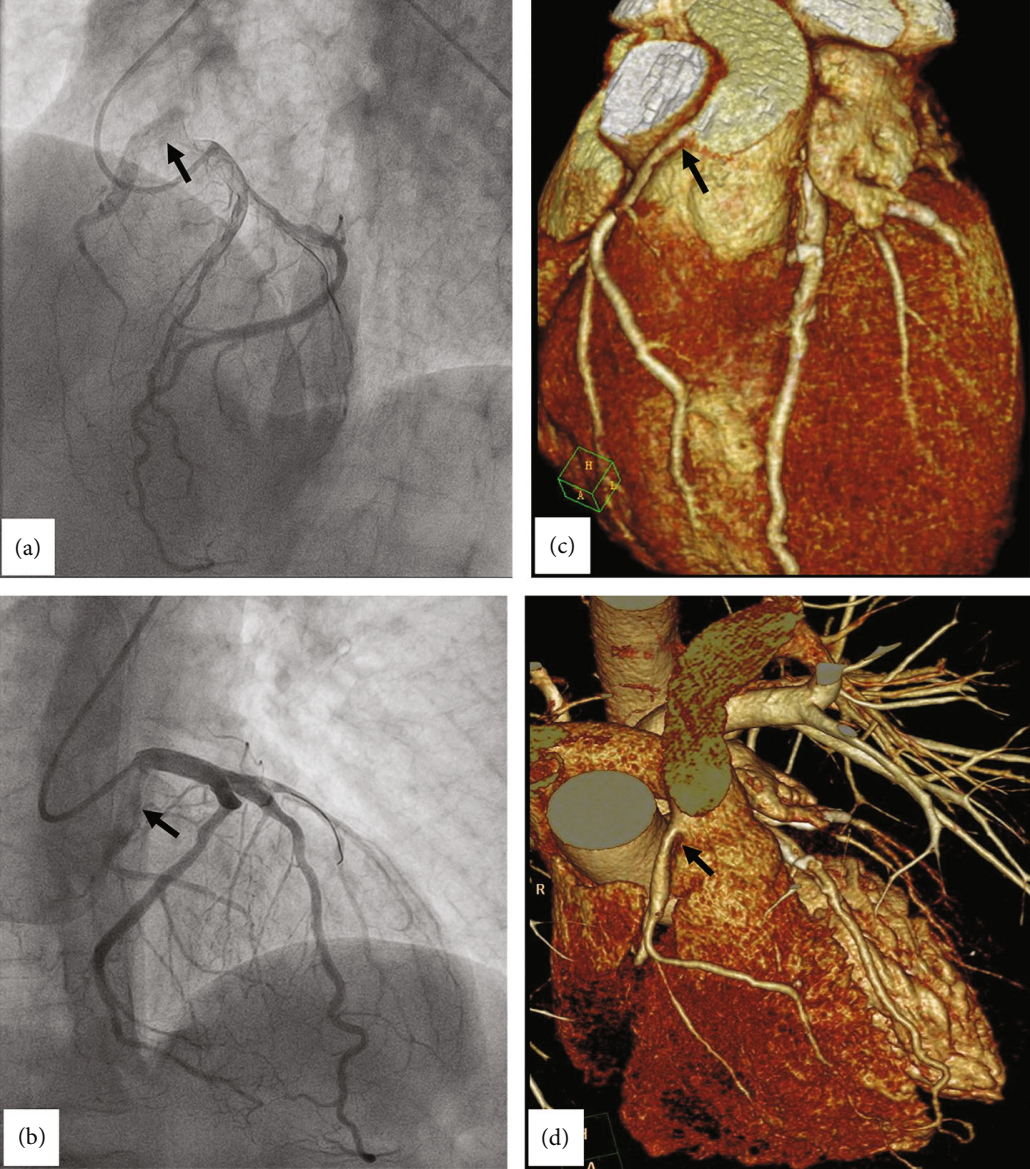
Coronary angiography, cranial views showing the retrograde filling of the right hypoplastic coronary artery toward the pulmonary artery (a, b). Corresponding projections from the computed tomography 3D reconstruction (the anomalous origin of the right coronary artery from the pulmonary artery is indicated by an arrow in c, d).

The patient was diagnosed as having an acute STEMI; he was referred to the Cath lab for emergency coronary angiography that showed a subocclusive stenosis of the proximal portion of the ramus intermedius artery (RIA) of the left coronary artery (LCA) (Figure 1b and Supporting Information 2: Video S2). Despite efforts and the use of multiple diagnostic catheters (Judkins Right, Right Coronary 3-dimensional, Amplatz Left 1), selective engagement of the right coronary artery (RCA) was not achieved. Aortography raised the suspicion of a coronary artery anomaly since it allowed the identification of collateral circulation between the left and right coronary arteries, even without showing a clear origin of RCA. Considering the EKG findings demonstrating no ST elevation in the inferior and right ventricular leads, together with the anatomical course of the RIA, we identified the RIA as the culprit vessel and proceeded with primary angioplasty. During contrast injections in LCA with a guide catheter (Extra Back-up 3), the extensive collateral circulation between the left and the right hypoplastic coronary arteries became more evident, especially from the septal branches of the left anterior descending artery (Figure 1b and Supporting Information 2: Video S2). Moreover, a retrograde filling from the right hypoplastic coronary artery toward the pulmonary artery (PA) emerged, strengthening the suspicion of ARCAPA (Figure 2a,b and Supporting Information 3: Video S3 and Supporting Information 4: Video S4). Therefore, primary angioplasty was performed on the RIA with the deployment of a biodegradable polymer biolimus-eluting stent and optimal angiographic result (Figure 1c and Supporting Information 5: Video S5). After the procedure, we observed complete symptom relief and the EKG showed regression of ST-segment elevation with T-wave inversion in lateral leads. To better assess the suspected anomalous origin of RCA, we performed a coronary computed tomography angiography (CCTA) 48 h after PCI. This confirmed the origin of the hypoplastic RCA from the PA (Figure 2c,d) with good patency of the DES on the RIA.

**Figure 3:**
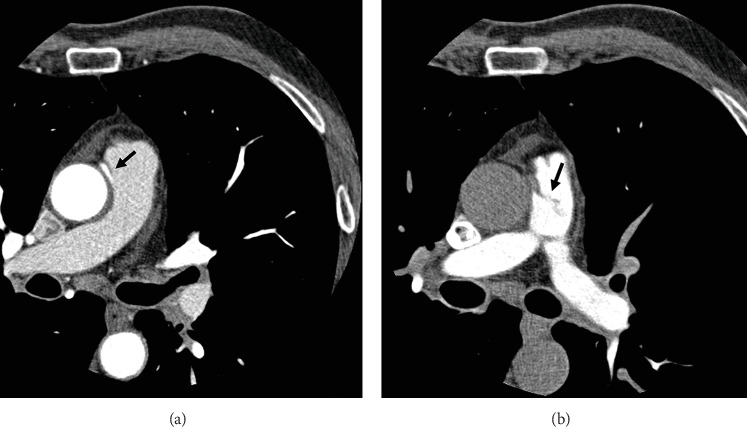
Computed tomography, axial plane arterial phase showing the anomalous origin of the right coronary artery from the pulmonary artery (arrow in a). In the venous phase, a retrograde filling toward the pulmonary artery is evident (arrow in b).

The anomalous origin was observed in the CCTA arterial phase (Figure 3a and Supporting Information 6: Video S6) while the retrograde filling of the PA became evident in the venous phase (Figure 3b). A diagnosis of ARCAPA was established. Given the absence of symptoms attributable to ARCAPA and the presence of a hypoplastic RCA, we chose a conservative strategy according to ESC 2020 guidelines [2]. The occurrence of an anomalous origin of the coronary artery led to an increase of about 20% (13 min) in door-to-balloon time when compared to the median time of 65 min recorded at our institution. Moreover, the identification of ARCAPA was associated with a radiation exposure time of 14 min and a contrast medium volume of 200 mL. The patient was discharged on Day 7 with dual antiplatelet therapy, beta-blockers, ACE inhibitors, statins, and lifestyle modification advice, in accordance with current ESC guidelines for the management of acute coronary syndrome. At the 12-month follow-up, no adverse events were reported, and the exercise stress test demonstrated normal functional capacity with no evidence of inducible ischemia.

## 3. Discussion

The clinical manifestations of ARCAPA and the timing of symptom onset are related to the development of intercoronary collateral circulation following the postnatal decline in PAP and PVR. The decrease of PVR and PAP leads to a backward blood flow into the PA, a phenomenon known as “intercoronary steal” (Figure 3b), that can lead to myocardial ischemia [3]. ARCAPA adult patients are asymptomatic in one-third of the cases; the remaining two-thirds may present with symptoms including stable angina, dyspnea, and heart failure [4]. If collateral coronary artery flow from the LCA provides blood supply to the right ventricular myocardium, patients may progress into adulthood without symptoms, and ARCAPA might become apparent due to atherosclerotic coronary artery disease or as an incidental finding [3]. In our case, mild RCA proximal stenosis and acute coronary origin from the PA might have reduced the steal phenomenon and symptoms.

The identification of the culprit lesion in an acute setting can be challenging in the presence of a previously unknown coronary artery origin anomaly, especially when the culprit lesion and the coronary artery with abnormal origin supply the same ventricular wall [5]. Selective engagement of the anomalous coronary artery often requires multiple catheters, prolonged door-to-balloon time, plus increased contrast volume and radiation exposure [6].

Moreover, the inability to engage the ostium of an anomalous coronary artery can lead to a wrong diagnosis of the culprit lesion [7]. ARCAPA should be suspected in the presence of RCA dilatation, retrograde flow through the RCA, direct flow between the RCA and PA, and extensive collateralization between the LCA and RCA [8]. However, it is important to consider not only abnormalities of origin, as in our case, but also abnormalities of course and termination in order to gain a complete understanding of coronary anatomy before proceeding to primary PCI [9]. In fact, the condition of the nonculprit vessels plays a key role in assessing the degree of hemodynamic support that may be required during PCI [10].

## 4. Conclusion

Our case highlights that coronary anomalies can increase the complexity in primary PCI, resulting in prolonged door-to-balloon time [6]. ARCAPA is an extremely rare condition; it is rarely promptly diagnosed in the acute coronary syndrome setting and can lead to an incorrect diagnosis of the culprit lesion [7]. It is imperative for interventional cardiologists to possess a comprehensive knowledge of various coronary artery anomalies, to maintain vigilance regarding the potential occurrence of such anomalies, and to implement effective management strategies for addressing them.

Patient's symptoms and multimodality imaging assessment, including coronary angiography, CCTA, and functional tests for myocardial ischemia, are essential for the definition of a treatment tailored to the patient's characteristics [4].

## Data Availability

The data that support the findings of this study are available on request from the corresponding author. The data are not publicly available due to privacy or ethical restrictions.

## Supporting Information

Supporting Information 1 Video S1: Echocardiogram, three- and four-chamber views showing lateral and inferolateral left ventricular wall hypokinesia.

Supporting Information 2 Video S2: Coronary angiography, caudal view showing subocclusive stenosis of the ramus intermedius.

Supporting Information 3 Video S3: Coronary angiography, cranial views: extensive collateral circulation between the left and right hypoplastic coronary arteries with retrograde filling toward the pulmonary artery (PA).

Supporting Information 4 Video S4: Coronary angiography, cranial views: extensive collateral circulation between the left and right hypoplastic coronary arteries with retrograde filling toward the pulmonary artery (PA).

Supporting Information 5 Video S5: Coronary angiography, caudal view showing optimal results after PCI of ramus intermedius.

Supporting Information 6 Video S6: Computed tomography, axial plane, arterial phase. Emergence of the right coronary artery from the pulmonary artery is noted.
